# A Novel SimpleProbe PCR Assay for Detection of Mutations in the 23S rRNA Gene Associated with Macrolide Resistance in Mycoplasma genitalium in Clinical Samples

**DOI:** 10.1128/JCM.01233-16

**Published:** 2016-09-23

**Authors:** Marianne Gossé, Hilde Lysvand, Brita Pukstad, Svein Arne Nordbø

**Affiliations:** a Department of Cancer Research and Molecular Medicine, Norwegian University of Science and Technology, Trondheim, Norway; b Department of Laboratory Medicine, Children's and Women's Health, Norwegian University of Science and Technology, Trondheim, Norway; c Department of Medical Microbiology, St. Olavs Hospital HF, Trondheim University Hospital, Trondheim, Norway; d Department of Dermatology, St. Olavs Hospital HF, Trondheim University Hospital, Trondheim, Norway; Wheaton Franciscan Laboratory

## Abstract

Macrolide-resistant strains of Mycoplasma genitalium are an increasing problem throughout the world, and the implementation of a rapid and sensitive assay for mutation detection to guide treatment is needed. Macrolide-resistant strains have been shown to contain base substitutions in positions 2058 and 2059 (Escherichia coli numbering) in region V of the 23S rRNA gene. In this study, we present a SimpleProbe PCR followed by melting curve analysis to differentiate between macrolide-resistant mutants and wild types. The assay was performed on 159 Mycoplasma genitalium-positive samples, and the results were compared with DNA sequencing. We also looked at the prevalence of macrolide-resistant strains in a Norwegian population. Of 139 samples characterized successfully by sequencing, 54 (39%) were wild types and 85 (61%) were mutants, consisting of 59 (42%) A2059G, 24 (17%) A2058G, 1 (1%) A2058T, and 1 (1%) A2059C mutation. The melting curve analysis correctly differentiated between wild-type and mutant strains in all cases, but it could not identify the different mutant types. The SimpleProbe PCR proved to be a simple, rapid, and reliable method for the detection of macrolide-resistant isolates of Mycoplasma genitalium in a clinical setting.

## INTRODUCTION

Mycoplasma genitalium is a small bacterium with the ability to cause urethritis in men and urethritis, cervicitis, and pelvic inflammatory disease (PID) in women (1–3). The recommended treatment for urethritis when the infectious agent is unknown (often called nonchlamydial-nongonococcal urethritis, or NCNGU) is 7 days of doxycycline (100 mg twice daily) (4) or 1 g azithromycin given as a single dose (5). The preferred treatment for uncomplicated infection with M. genitalium in Scandinavia is 1.5 g azithromycin given over a period of 5 days, where 500 mg is given on day 1 and 250 mg is given on days 2 to 5 (4, 6). This prolonged treatment is preferred to avoid development of macrolide resistance (7). Recent studies show the emergence of resistant types of M. genitalium in many parts of the world (8–10). The most common mutations causing macrolide resistance are found in domain V in the 23S rRNA gene in positions 2058 and 2059 (Escherichia coli numbering) (11). A2058G and A2059G are the most common mutations reported, followed by A2058T and A2058C (10, 12). The second-line antibiotic following treatment failure with azithromycin is moxifloxacin, a fluoroquinolone (6). However, several reports of resistance to this group of antibiotics have also been published (13, 14).

With increasing prevalence of resistant strains, access to the assessment of macrolide sensitivity becomes important. The gold standard for detecting mutations in the 23S rRNA region is DNA sequencing, but the method is too laborious, time-consuming, and expensive for routine analysis. Touati et al. presented a real-time PCR using fluorescence resonance energy transfer (FRET) combined with melting curve analysis (15) to detect mutations. Twin et al. utilized high-resolution melting curve analysis (16). Wold et al. presented a method using PCR with a TaqMan probe and polymorphism-specific forward primers (17), although only for specimens that had a cycle threshold (*C_T_*) of less than 32, thereby excluding samples with a lower organism load. PCR followed by pyrosequencing has also been presented (12), and recently, Kristiansen et al. presented a 5′-nuclease genotyping assay for this purpose (18).

The method presented in this study is a SimpleProbe real-time PCR followed by melting curve analysis. This method uses a single probe labeled with a self-quenching fluorophore (19). Different mutations in the gene segment cause different melting temperatures and enable differentiation between wild-type and mutant strains.

Our aim was to develop a simple and sensitive method for diagnostic use to differentiate between the macrolide-susceptible wild type and mutant types of M. genitalium without the need for nucleotide sequence determination. The method has been used for the detection of other microbes (20, 21) but, to our knowledge, has never been applied to M. genitalium mutation detection. The study also aimed to present the prevalence of macrolide-resistant M. genitalium isolates in a Norwegian population.

## MATERIALS AND METHODS

### Sample collection and primary screening.

M. genitalium-positive samples were collected from the Department of Medical Microbiology at St. Olav's Hospital, Norway, between February 2015 and May 2015. The majority of specimens came from outpatients not attending sexually transmitted disease clinics. Samples had undergone DNA extraction and primary testing within 3 days after sampling before being frozen. All samples were obtained for study purposes and analyzed by SimpleProbe PCR in June 2015. DNA sequencing analysis were performed during the period September to November 2015. Primary screening was performed using a CFX96 real-time system C1000 thermal cycler (Bio-Rad Laboratories Inc., Hercules, CA, USA) and FTD urethritis basic kit (Fast-Track Diagnostics Ltd., Esch-sur-Alzette, Luxembourg) for the simultaneous and quantitative detection of Neisseria gonorrhoeae, Chlamydia trachomatis, and M. genitalium. The FTD urethritis basic kit is a multiplex real-time PCR with probes targeting the adhesin (*mgpB*) gene of M. genitalium, the cryptic plasmid ORF8 gene of Chlamydia trachomatis, and the *opaK* gene for the opacity protein of Neisseria gonorrhoeae. Murine cytomegalovirus is used as an internal control and detected by a probe targeting an intergenic region of this virus. The sensitivity of the M. genitalium assay is approximately 1,000 copies/ml according to the manufacturer. After excluding test-of-cure samples taken within 3 months and samples taken from different locations on the same patient, 159 primary samples were included in this study (Table 1). When samples taken from more than one location on the same patient were present, cervical swabs were preferred over vaginal swabs, vaginal swabs were preferred over urethral swabs, and urethral swabs were preferred over urine samples. The samples comprised 65 first-void urine samples (FVU) from men and 94 flocked swabs (88 swabs from women and 6 urethra swabs from men) in universal transport medium (UTM; Copan Italia S.P.A., Brescia, Italy). Swabs obtained from women were either vaginal (65%) or endocervical (35%). DNA was extracted from 1 ml urine and 200 μl transport medium from swabs using NucliSENS easyMAG (bioMérieux SA, Marcy l'Etoile, France). The manufacturer of the kit provided a validated formula for the direct quantification of the organisms detected in the samples. Extracted DNA was stored at −20°C until further processing. Wild-type G37 and strains with A2058G, A2058T, A2058C, and A2059G mutations were kindly provided by J. S. Jensen (Statens Serum Institut, Copenhagen, Denmark).

**TABLE 1:** Patient demographics showing number of samples and distribution of specimen type

Parameter	Male	Female	Total
No. of samples	71	88	159
Median age (yr; range)	28 (16–54)	24 (17–64)	26 (16–64)
Sample type (no. [%])			
Urine	65	0	65 (41)
Urethra swabs	6	0	6 (4)
Cervical/vaginal swabs	0	88	88 (55)

### SimpleProbe real-time PCR and melting curve analysis.

The reaction mixture for each sample consisted of 10 μl SsOAdvanced universal probe supermix (Bio-Rad Laboratories Inc., Hercules, CA, USA), 1 μl of forward primer Mg23S S (2 μM), 1 μl of reverse primer Pr f Sp mis (10 μM), 1 μl SP wild-type probe (10 μM; TIB Molbiol, Berlin, Germany), 2 μl molecular-grade water, and 5 μl DNA template. Primers and probe sequences are presented in Table 2. The amplification commenced at a single step of 95°C for 3 min, followed by 50 cycles at 95°C for 15 s and 55°C for 30 s, using a CFX96 real-time system C1000 thermal cycler (Bio-Rad Laboratories Inc., Hercules, CA, USA). After amplification, the melting curve analysis started at 45.0°C and ended at 75.0°C, with an increment of 0.5°C every 5 s. The sensitivity of the SimpleProbe assay was calculated to 6 copies per PCR using a 10-fold dilution series of AmpliRun Mycoplasma genitalium DNA control (Vircell, Granada, Spain) at 12,500 copies/μl.

**TABLE 2:** Primers and probe for melting curve analysis by the SimpleProbe assay and the positions of the primers according to 23S rRNA gene

Primer name	Sequence^*a*^	23S rRNA gene sequence position
Forward primer Mg23S S	5′-GGTTAAAGAAGGAGGTTAGCAATTT-3′	1850–1874
Reverse primer Pr f Sp mis	5′-AGCTACAGTAAAGCTTCACTGGG-3′	2090–2068
SP wild-type probe	5′-CGCA-XI-ACGGGACGGAAAGACC-3′	2050–2069

a The reverse primer intentionally contains one mismatch to avoid folding of the amplicon, and it contains the motif ACGGG (under the probe) and CCCGT (at the 3′ end of the primer). The calculated melting temperature for the folded structure is 84°C. The folded PCR product will compete with probe binding and cause low signals and secondary lower melting temperature peaks. XI in the probe sequence is the position of the SimpleProbe 519 labeling reagent, with X indicating any modified residue and I indicating an analogue for d-inosine as a non- or low-binding base moiety.

### DNA sequence determination.

To determine the 23S rRNA gene sequences, all samples were DNA sequenced after melting curve analysis. Amplification prior to sequencing was performed by a T100 thermal cycler (Bio-Rad Laboratories Inc., Hercules, CA, USA) with the same reaction mixture as that for the SimpleProbe real-time PCR, excluding the probe at an annealing temperature of 63°C. The higher annealing temperature reduces unspecific amplification in a mixture where there is no probe, as the specific binding of the probe in the SimpleProbe reaction helps to ensure specific detection at a lower annealing temperature. The analysis of the size of the amplicon and DNA concentration was performed using an Agilent 2100 Bioanalyzer (Agilent Technologies Inc., Santa Clara, CA, USA), and product cleanup was achieved with a rapid PCR cleanup enzyme set (New England BioLabs Inc., Ipswich, MA, USA). A GenomeLab DTCS quick start kit (Beckman Coulter Inc., Brea, CA, USA) was used for dye terminator cycle sequencing of the 241-bp amplicon from the 23S rRNA gene with the forward primer Mg23S S. The sequence products were analyzed by a CEQ8800 genetic analysis system (Beckman Coulter Inc., Brea, CA, USA).

### Statistics.

Cohen's Kappa coefficient for sensitivity, as well as 95% confidence intervals (CI), were calculated for measurement of interrater agreement of the tests.

### Ethics.

As the study was considered an internal quality control study, approval from the Regional Ethics Committee was not required, although it was contacted to confirm this. An approval from the internal Data Protection Official at St. Olavs University Hospital was required, and approval was received.

## RESULTS

### Test sensitivity.

In the primary screening test, the range of the bacterial load from swabs and urine samples was 3.39 × 10^2^ to 1.51 × 10^7^ copies/ml (median, 8.47 × 10^3^ copies/ml) and 5.87 × 10^3^ to 2.40 × 10^6^ copies/ml (median, 1.34 × 10^4^ copies/ml), respectively. The sensitivity of the genotyping methods is shown in Table 3, where the lowest bacterial load in specimens yielding a successful melting curve analysis and sequencing results are listed. Of the 159 patients with M. genitalium-positive samples that later underwent both SimpleProbe PCR analysis and DNA sequencing, 17 (11%) were negative by both methods. Of the remaining 142 samples, 139 were positive in SimpleProbe PCR and 139 yielded a positive result when DNA was sequenced. Six samples yielded positive results in only one of the two tests, and 136 samples were positive by both tests. The kappa value was 0.91 (with 95% CI of 0.74 to 1.08). Among the 136 specimens with positive results, five samples had no amplification curve in the SimpleProbe assay. However, melting curve analysis could be interpreted, and the result correlated with the DNA sequencing. The test sensitivity for both methods was 87% (CI, 0.817 to 0.923).

**TABLE 3:** Lowest bacterial load in swab and urine specimens yielding a successful melting curve analysis and DNA sequencing result

Assay	Bacterial load (copies/ml) by sample type^*a*^	
	Swab	Urine
SimpleProbe PCR assay		
Lowest copy no. yielding a positive result	6.99 × 10^2^	1.31 × 10^2^
Lowest copy no. when all samples with higher bacterial load yielded a positive result	5.99 × 10^3^	1.31 × 10^2^
DNA sequencing		
Lowest copy no. yielding a positive result	4.66 × 10^2^	1.02 × 10^2^
Lowest copy no. when all samples with higher bacterial load yielded a positive result	5.99 × 10^3^	7.98 × 10^3^

a All swabs containing more than 5.99 × 10^3^ copies/ml and all urine samples containing more than 1.31 × 10^2^ copies/ml yielded a positive result by the SimpleProbe assay. The corresponding results for the DNA sequencing were 5.99 × 10^3^ copies/ml and 7.98 × 10^3^ copies/ml, respectively.

### Melting curve analysis.

**FIG 1:**
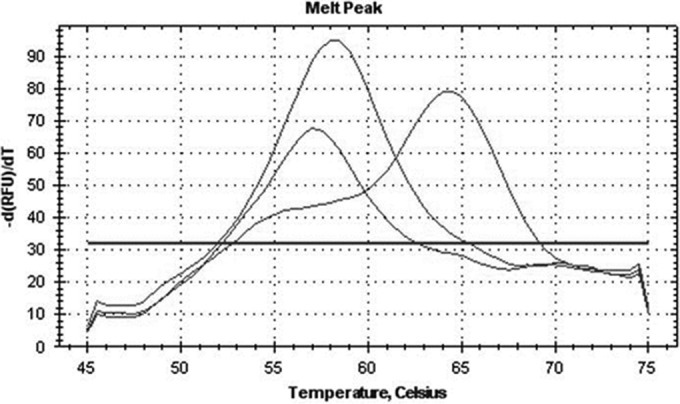
Example of melting temperatures of a wild-type strain (64.5°C) and strains with A2058G (58°C) and A2059G (57°C) mutations. The clear distinction of melting temperatures between wild-type and mutant strains is shown.

The wild types had a melting temperature ranging between 64°C and 65°C, whereas the mutants had melting temperatures ranging between 57°C and 59°C (Fig. 1). Samples with a high *C_T_* value from the routine testing had a tendency toward higher melting temperatures, but all were within the given range.

### Distribution of M. genitalium 23S rRNA mutations.

Of the successfully DNA sequenced samples, there were 54 (39%) wild types and 85 (61%) mutants (Table 4). Four types of mutations were present: 59 A2059G (69%), 24 A2058G (28%), 1 A2059C (1%), and 1 A2058T (1%). Melting curve analysis and sequencing results for the 136 samples that were positive by both methods showed complete concordance with each other (Table 4). Sequencing data contained no evidence that multiple templates were present in any of the samples.

**TABLE 4:** Concordance of SimpleProbe PCR and DNA sequencing for determination of 23S rRNA gene mutations

SimpleProbe PCR	DNA sequencing^*a*^					
	WT	23S rRNA mutant				ND
		A2059G	A2058G	A2059C	A2058T	
WT	52					1
23S rRNA mutants		58	24	1	1	2
ND	2	1				

a ND, not determined.

## DISCUSSION

In this study, we report a large number (61%) of patient samples with mutations in region V of the 23S rRNA gene of M. genitalium. The most commonly reported mutations to cause macrolide failure (11, 22), A2059G and A2058G (12, 16, 17), represented 42% and 17%, respectively, of the 139 positive samples. We also report the presence of the less common mutations A2059C and A2058T. A2059C has been reported in Australia (16), France (23), and England (8), but to our knowledge it has not been reported in Scandinavia before.

The range of the bacterial load from swabs was 3.39 × 10^2^ to 1.51 × 10^7^ copies/ml and 5.87 × 10^1^ to 2.40 × 10^6^ copies/ml for the urine samples in the primary screening. The better sensitivity for the urine samples correlates well with the fact that the DNA extraction volume was five times larger for the urine samples (1,000 μl) than the swab specimens (200 μl).

In this study, there was a complete correlation between the SimpleProbe assay and DNA sequencing for 136 (86%) of the samples. Kappa analysis of the two methods gave a score of 0.91 (95% CI, 0.74 to 1.08), indicating a strong agreement of the two methods. The remaining 23 (14%) samples were negative in either one or both tests, but none yielded contradictory results. All samples had been frozen and thawed between every set of processing. Hence, the quality of the DNA in the samples may have been reduced after the first PCR run (24). Five of the positive samples had no amplification curve in the SimpleProbe PCR but had a melting curve that correlated with the sequencing results. A possible explanation for this is that for the SimpleProbe assay, the annealing temperature at 55°C had been optimized with respect to the melting curve analysis, whereas the annealing temperature in the conventional PCR prior to the sequencing reaction had been optimized to 63°C. These samples were probably at the limit of detection for the method, and it is likely that with a few more amplification cycles we would have achieved an amplification curve. This optimization of annealing temperatures might also explain the reason why three samples turned out negative in the SimpleProbe assay but positive when DNA was sequenced. All specimens with a bacterial load of more than 6,000 copies/ml in the primary screening test were positive in both the SimpleProbe assay and by DNA sequencing.

The melting temperature of the macrolide-resistant strains varied between 57°C and 59°C. However, the differences between the melting temperatures of these strains were too small to distinguish between the different mutations. In addition to having overlapping melting temperatures, the individual samples could vary by 0.5°C from run to run. The mutants were, however, easy to distinguish from the wild types, with melting temperatures of 64 to 65°C. Therefore, DNA sequencing was the method of choice to distinguish the different mutant types from each other. We observed that the melting temperature had a tendency to be higher as the sample concentration decreased (higher *C_T_* values). The definition of melting temperature is when 50% of the opposing strands are disassociated, and it is possible that bacterial concentration influences this balance.

Some researchers have reported urine samples to have higher sensitivity than urethral swabs from males for the detection of Chlamydia trachomatis and M. genitalium (25), and this practice is recommended by the Norwegian Institute of Public Health (26). This is the reason for the small number of urethral swabs evaluated, as urethral swabs from males in our health region usually are collected only in cases where infection with Neisseria gonorrhoeae is suspected.

Screening for M. genitalium in asymptomatic patients is controversial. The high sensitivity of nucleic acid amplification tests may result in the detection of low-grade infections that have the potential to clear spontaneously or would never cause any harm to the patient (27). Unlike for Chlamydia trachomatis, not much is known about the natural history of M. genitalium, and as M. genitalium is becoming increasingly harder to treat, the cost versus benefit of screening needs to be considered carefully. Macrolide resistance is an increasing problem throughout the world (8, 9), and there is concern about the increased use of fluoroquinolones. The new fluoroquinolones potentially can cause severe adverse events and are rarely first-line agents. This important class of antibiotics should be employed judiciously (28). Several studies have shown emerging fluoroquinolone resistance (9, 29), and there is a need for international guidelines for appropriate treatment.

The large number of macrolide-resistant M. genitalium strains verified in this study (61%) is of great concern. To avoid unnecessary use of macrolides for the eradication of M. genitalium, a pretreatment mutation detection would be beneficial. The SimpleProbe PCR combined with melting curve analysis is a rapid, sensitive, and reliable method to distinguish wild types from macrolide-resistant mutants. It can be implemented easily in a routine laboratory setting and allows for detection of at least four mutations in the same locus on the 23S rRNA gene. Further studies are needed to evaluate the usefulness of this method in a clinical setting.
